# Evaluation of efficacy, safety, pain perception and health-related quality of life of percutaneous ethanol injection as first-line treatment in symptomatic thyroid cysts

**DOI:** 10.1186/s12902-015-0069-3

**Published:** 2015-11-26

**Authors:** Jordi L. Reverter, Núria Alonso, Marta Avila, Anna Lucas, Dídac Mauricio, Manel Puig-Domingo

**Affiliations:** Department of Endocrinology and Nutrition, Germans Trias i Pujol Health Science Research Institute and Hospital, CIBER of Diabetes and Associated Metabolic Diseases (CIBERDEM), Universitat Autònoma de Barcelona, Carretera de Canyet s/n, 08916 Badalona, Spain; Department of Pathology, Germans Trias i Pujol Health Science Research Institute and Hospital, Universitat Autònoma de Barcelona, Badalona, Spain

**Keywords:** Thyroid cysts, Percutaneous ethanol injection, Health-related quality of life, Pain

## Abstract

**Background:**

To evaluate the efficacy, safety, pain perception and health-related quality of life (QoL) of percutaneous ethanol injection treatment (PEIT) as an alternative to thyroid surgery in symptomatic thyroid cysts.

**Methods:**

Thirty consecutive patients (46 ± 10 years; 82 % women) with symptomatic benign thyroid cysts relapsed after drainage were included. In all cases, cytology prior to treatment, maximum cyst diameter and volume were determined. PEIT was conducted using the established procedure, and the volume of fluid removed and pain perceived by the patient were assessed. In each procedure, the volume of alcohol instilled was <2 ml. After follow-up, final cyst diameter and volume were determined and the persistence of symptoms and QoL were assessed by a questionnaire (SF-36).

**Results:**

Mean symptom duration was 10 ± 20 months. A single session of PEIT was required to complete the procedure in 45 % of patients, two in 31 % and three in 13 %. Mean initial maximum cyst diameter was 3.5 ± 1.0 cm and mean extracted liquid volume 61 ± 36 ml. During PEIT, 39 % of patients experienced virtually no pain, 43 % mild pain and 17 % moderate pain. No complications of PEIT were observed. After 12.1 ± 1.4 months of follow-up, cysts were reduced more than 70 % in volume in 86.3 % of patients, more than 80 % in 61.9 % and more than 90 % in 42 %. On the health-related QoL SF-36 questionnaire, patient scores 6 months post-PEIT did not differ significantly from those of the healthy Spanish population. With respect to cosmetic complaints or local symptoms of compression, PEIT-treated patients presented an initial score of 22 ± 8 and 13 ± 5 after treatment (*p* < 0.05).

**Conclusions:**

In our experience, percutaneous ethanol injection has prove to be an effective, safe and well-tolerated first-line treatment of symptomatic thyroid cysts.

## Background

Thyroid nodules are common in the general population with a prevalence of palpable lesions between 3 and 10 % [1]. Moreover, non-palpable ultrasound-detected thyroid nodules are present in 20 to 60 % of healthy individuals [2]. Thyroid cysts are characterised by their liquid contents and are mostly benign [3]. After percutaneous drainage, most cystic lesions (around 80 %) refill and enlarge over time. In cases of recurrent thyroid cysts producing aesthetic complaints or compressive symptoms, surgery has been the first-line treatment to date [4]. Since the late 1990s, sonography-guided percutaneous ethanol injection treatment (PEIT) has emerged as a safe and effective conservative alternative to surgical excision [5]. The injection of 95–99 % ethanol into the cyst cavity induces thrombosis of small vessels and coagulative necrosis surrounded by interstitial oedema and granulomatous inflammation, followed by fibrosis, shrinkage and reduction in the volume of the lesion [5, 6]. A randomised study from Bennedbaek and Hegedüs reported a significant 82 % volume reduction in the PEIT group (*n* = 33) compared to 18 % in the saline treated group (*n* = 33) [7], with few complications, thereby providing data permitting this technique to be considered as a reliable alternative to thyroid surgery. In this respect, recent guidelines in the USA and Europe [2] state that PEIT is a clinically-effective, non-surgical option for repeatedly drained recurrent thyroid cysts. However, there is a paucity of reports in the literature from few centres describing the outcomes of this technique for avoiding surgery in these patients when it is introduced in systematic clinical practice.

Furthermore, as a recent review of the Cochrane Collaboration established, previous studies on this technique did not provide information on health-related quality of life (QoL) and the authors of the review thus suggested that this important measure for the patient should also be a primary end-point in future trials. [8].

Herein, we reported our results on effectiveness, safety, pain perception and health-related QoL in a group of patients with symptomatic recurrent thyroid cysts after PEIT.

## Methods

Thirty consecutive patients treated at the outpatient clinic of the Endocrinology and Nutrition Department of the Germans Trias i Pujol Universtity Hospital, Badalona, in 2013 and referred for evaluation of a cervical nodule or nodular goiter were included. A case was considered for PEIT if it met all the following selection criteria: 1) age >18 years; 2) normal thyroid function tests; 3) no major comorbidities; 4) no history of neck irradiation; 5) cystic or predominantly cystic (>80 % cystic component) thyroid nodule; 6) compressive symptoms or aesthetic complaints; and 7) voluntary patient decision to undergo PEIT instead of open surgery after careful explanation of the procedure. The study was conducted in accordance with the Declaration of Helsinki and was approved by the local Human Research Ethics Committee of the Hospital Germans Trias i Pujol. All participants gave their written informed consent.

In all cases, a medical history was taken and physical examination performed and blood samples were obtained for analytical determinations. Serum free thyroxine (fT4) and TSH were measured by electrochemiluminescence immunoassay (Siemens^©^, Los Angeles, CA, USA).

### Ultrasound examination

**Fig. 1:**
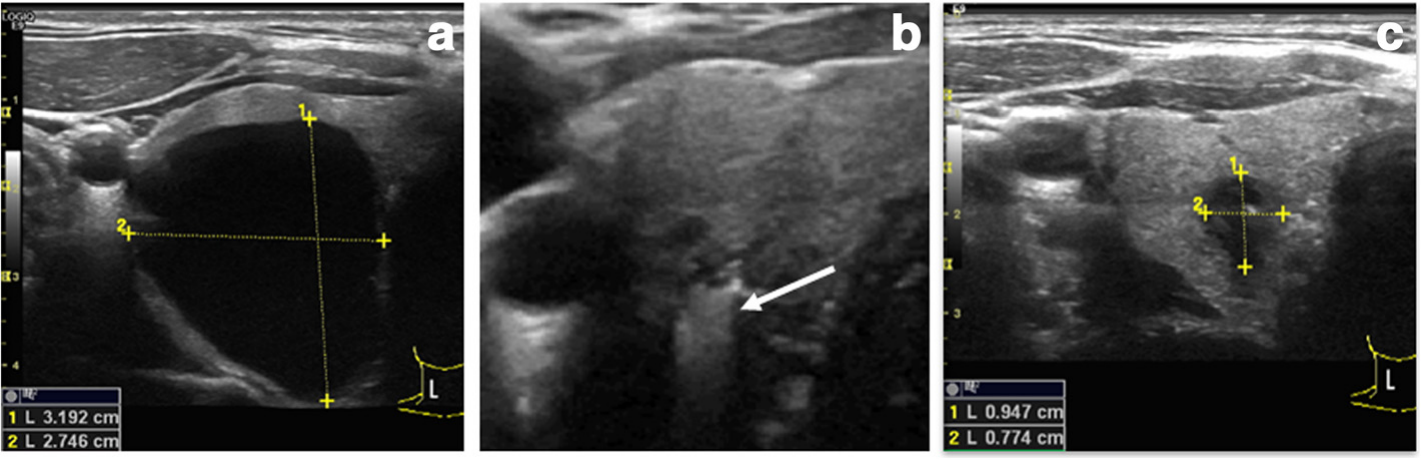
Transverse ultrasound scans of one cyst before (panel **a**), during (panel **b**) and after (panel **c**) percutaneous ethanol injection treatment (PEIT). During PEIT, cystic lumen was filled with instilled ethanol (*arrow*). Panel **c** shows marked decrease in size

An echography examination was performed in each patient by an experienced operator (JLR) using a 12–15 MHz linear transducer device. Morphological evaluation included description of the thyroid echostructure and measurement of the diameters and ultrasound characteristics of each detected nodule (Fig. 1, panel a). Volumetric assessment of the nodules was based on the use of an ellipsoid model [9]. With this rotating ellipsoid model, the height, width and depth of each nodule were measured and multiplied. The obtained result was then multiplied by the mathematical constant or correction factor 0.524 [10]. Fine-needle aspiration (FNA) cytology was performed in all cases to obtain samples from all nodules >1.0 cm in diameter and those between 0.5 and 1.0 cm if they presented suspicious ultrasound characteristics according to the American Society of Ultrasonography [11] such as the presence of microcalcifications, irregular borders, increased central flow on Doppler examination, taller than wide diameter, hypoechogenicity and absence of halo. In the case of cysts, FNA was performed from the capsule or solid part of the nodule and for drainage of the cyst contents, and cytological analysis was performed. Patients were offered PEIT if the cyst presented significant regrowth after the first drainage with compressive symptoms or aesthetic complaints and cytological analysis was benign and preferred not to undergo thyroid surgery after extensive explanation of the procedure.

### Percutaneous ethanol injection treatment procedure

Positions of the patient and operator were similar to those of the FNA procedure, with the patient lying with their neck in hyperextension. After skin sterilisation, and under ultrasound guidance, a 21 G needle mounted on a connector with a line extension and a 20 ml syringe were used to empty the contents of the cyst. Subsequently, 0.5 ml of 5 % lidocaine was infused into the cyst cavity and, after a 2 min wait, 99 % ethanol was injected with a slow movement of the needle to reach the major part of the inner face of the cyst capsule (Figure 1, panel b). The patient was instructed to signal any feeling of pain to prevent the spread of alcohol into the neck structures. The amount of ethanol injected was 50 % of the volume of liquid extracted with a maximum of 2 ml. The needle tip was constantly visualsed during the procedure. In no case were more than 2 ml injected. The ethanol was not re-extracted and patients were discharged after a 20–30-minute observation period.

### Follow-up

Patients were followed up weekly for 1 month, monthly for 3 months and every 3 months thereafter. Ultrasound examination was performed at each appointment to evaluate the characteristics and volume of the residual lesion (Fig. 1, panel c).

In cases of symptomatic cyst regrowth confirmed by echography, a new PEIT was performed with the same protocol followed by weekly appointments. This procedure was repeated until permanent and significant cyst reduction was achieved or the patient decided not to repeat the PEIT procedure. No limits were set in the number of PEIT procedures.

### Response parameters

In each patient, the percentage of volume reduction was calculated by the formula [(V1 − V2)/V1] × 100 in which V1 is the initial cyst volume and V2 the final cyst volume.

Patients were requested to grade the sensation of pain suffered with the alcohol injection immediately after the procedure, using a visual scale of 10 cm between the absence of pain (0) and excruciating pain (10) in which each 1/3 of the scale corresponded, from the lowest to the highest, to the following categories: mild pain, moderate pain and severe pain.

### Health-related quality of life

The Spanish version of the SF-36 questionnaire provided online by www.sf-36.org/ was administered to each patient 6 months after the last PEIT. The SF-36 questionnaire is a multipurpose, short-form health survey, commonly used to evaluate patients’ health-related QoL in clinical practice. Eight domains are assessed in this questionnaire, including physical functioning, role limitation due to physical problems, bodily pain, general health, vitality, social functioning, role limitation due to emotional problem, and mental health. The eight health domains were used to provide a physical component summary (PCS) and a mental component summary score (MCS). Interpretation of the SF-36 Health Survey was greatly simplified with the norm-based scoring (NBS) of its health domain scales and component summary measures. In NBS, each scale is scored to have the same average (50) and the same standard deviation [10], signifying that each point equals one-tenth of a standard deviation [12]. For all scales and summary measures mean group scores below 47 can be interpreted as being below the average range for the general population.

With a view to assessing the specific symptoms of neck cysts derived, we created a domestic non-validated questionnaire including ten items related to the most frequent goitre symptoms described in the literature. These symptoms were related to visible enlargement of the neck (cosmetic complaint), feeling of pressure in the neck, throat pressure, sore throat, pain in the throat radiating to the ears, difficulty in swallowing, throat clearing often, shortness of breath and hoarseness. Each item was scored between 1 (none of the time), 2 (a little of the time), 3 (some of the time), 4 (most of the time) and 5 (all of the time), and the final score was the sum of the ten items. Thus, a score could range from ten (absence of symptoms) to 50 (patient’s worst feeling of symptoms). Compressive symptoms on the trachea were evaluated by chest X-ray.

### Statistical analysis

Continuous variables were expressed as mean ± SD or median (interquartile range) and categorical variables as percentages. Student’s t-test was used for comparisons between continuous variables and Pearson’s correlation test for correlation analyses. A *p* value < 0.05 was considered statistically significant and correlations were considered significant for Pearson’s correlation coefficient if ≥0.25. For comparison of categorical variables, the chi-square test or Fisher’s exact test were used where appropriate. Data analyses were made using the Statistical Package for Social Sciences (SPSS^©^, Chicago, IL, USA) for Windows®, Version 15.0.

## Results and discussion

Thirty subjects with symptomatic thyroid cysts (46 ± 10 years; 82 % women) were included in the protocol. Mean duration of symptoms was 10 (1–36) months and, in all cases, TSH and fT4 were within the normal range. FNA cytology was benign in all samples obtained (haemorrhagic-cystic lesion, colloid cyst or non-malignant lesion) from the predominantly cystic nodules and the other nodules with sonographic criteria for FNA cytology [13]. Mean maximum cyst diameter before drainage was 3.5 ± 1.0 cm with a calculated initial volume of 18.2 ± 15.5 ml (median 14.5 ml). Mean total volume extracted from the cysts in all procedures performed was 61.0 ± 36.5 ml (2–265 ml).

The required number of PEIT for permanent reduction in cysts and a symptom-free situation was one in 45.5 % of cases, two in 31.8 % and three in 13.6 %. Two patients with the largest cysts in this series (total volume extracted: 145 and 265 ml) were treated four and six times, respectively. Initial calculated cyst volume was significantly greater in the group of patients who required three or more PEIT procedures compared with those treated with one or two PEITs (29.6 ± 25.5 ml *vs*. 15.5 ± 10.6 ml, respectively; *p* < 0.05).

In all cases requiring more than one treatment, cyst recurrence was observed during the first month post-treatment, both for the first PEIT and subsequent procedures. No delayed recurrences were observed.

The sensation of pain reported during PEIT was virtually absent (39.1 %), mild (43.5 %) or moderate (17.4 %). In all cases, this was a transient sensation of pain with relief within minutes. No patient felt intense pain and none suffered other complications.

After 12.1 ± 1.4 months of follow-up mean calculated cyst volume reduction was 85.9 ± 14.7 %. In all cases, cyst volume was reduced more than 50 % from the basal. Cysts were reduced more than 70 % in volume in 86.3 % of patients, more than 80 % in 61.9 % and more than 90 % in 42 %. Final mean maximum thyroid cysts diameter was 1.3 ± 0.6 cm. Total disappearance of the cysts was observed in three cases.

Regarding the characteristics of cystic lesions, 19 of the cysts were purely cystic nodules, and 11 mixed or predominantly cystic nodules (with a <20 solid component). No differences were observed between cystic and mixed nodules in calculated baseline volume (19.7 ± 16.1 vs 14.8 ± 8.7 ml, respectively; *p* = 0.3), calculated final volume (1.6 ± 2.6 vs. 1.9 ± 1.6 ml, respectively; *p* = 0.7) and volume reduction (88.2 ± 12 vs. 80.3 ± 17.5 % respectively; *p* = 0.2). The number of PEIT sessions required was also similar (one in 47.3 vs. 50.0 %, *p* = 0.8; in purely cystic and mixed cysts, respectively).

Table 1 summarizes the final volume observed and the volume reduction according to the calculated initial volume of the cysts. No significant differences were observed between cysts >15 ml in the calculated final volume (*p* = 0.7). As expected, volume reduction was significantly (*p* = 0.002) greater in nodules >15 ml (78.1 ± 15.8 %) compared to those <15 ml (93.7 ± 7.5 %), *p* = 0.002.

**Table 1:** Cyst volume results

	<15 ml (*n* = 15)	>15 ml (*n* = 15)	*P* value
Baseline volume (ml)	8.4 ± 4.1	28.8 ± 13.1	0.001
Final volume (ml)	1.6 ± 1.5	1.9 ± 2.9	0.7
Volume reduction (%)	78.1 ± 15.8	93.7 ± 7.5	0.002

Results on calculated cyst volume and percentage of volume reduction in patients treated with percutaneous ethanol injection treatment (PEIT) according to their initial volume

In the health-related QoL SF-36 questionnaire, norm-based PCS [52.4 (4.4)] and MCS [49.3 (4.8)] scores of the patients 6 months after PEIT did not significantly differ from those of the healthy Spanish population [14].

With respect to specific questions addressing to cosmetic complaints or local symptoms of compression, PEIT-treated patients presented an initial score of 22 ± 8 and after treatment 13 ± 5 (*p* < 0.05) with 47 % of cases reporting a score of ten, i.e. absence of symptoms.

In the correlation analysis, a significant relationship observed were between calculated initial cyst volume and evolution time (*r* = −0.4, *p* = 0.02) and between baseline calculated volume and volume reduction (*r* = −0.1, *p* = 0.04).

## Discussion

In our experience, percutaneous ethanol treatment is a safe, well-tolerated and effective procedure for the treatment of symptomatic benign thyroid cysts, achieving relief or the disappearance of the aesthetical complaints and/or compressive symptoms in all treated patients. Moreover, patients who underwent this technique showed a health-related quality of life related similar that of the normal population according to a validated questionnaire.

Since the introduction of PEIT for thyroid cysts in clinical practice, some groups from Italy [15–18], Denmark [7] and South Korea [19, 20] reported a significant high success rate (more than 80 %) with no significant side effects. Based on these results, the major endocrine society guidelines state that treatment of recurrent benign thyroid cysts with ethanol in experienced hands is a clinically effective, non-surgical option for purely or predominantly cystic thyroid nodules that recur after repeat aspirations [2, 21]. The results obtained in our group of treated patients after the PEIT procedure was introduced as a regular practice at our hospital concur with those good results report in terms of efficacy and safety, with more than 70 % volume reduction in more than 85 % of patients and the disappearance of aesthetic complaints or compression in almost half of cases and significant relief in the rest.

In our protocol, we offered PEIT after the first cyst drainage. With this approach, the benign nature of the lesion was confirmed by cytological examination of the samples and the refilling of cysts that occurred in all cases in this series. The number of PEIT sessions required ranged from one to six depending on initial cyst size and volume. However, the majority of cases (87.3 % in our series) were resolved with the first or second procedure and whether more than two treatments were required depended on initial cyst volume. In our opinion this feature is important in order to discuss with the patient before the procedure the eventual needing of more than one or two PEITs.

The volume of ethanol used in PEIT is a matter of debate. The reported amounts of ethanol administered ranged from 35 to 100 mL depending on the authors [6, 7, 16, 19], with a recommended maximum single infusion of 10 mL, although treatments with the instillation of up to 30 mL have been reported [22]. In our protocol, we decided to use a maximum ethanol volume of less than 2 mL in all cases. We, like others [23], believe this cautious approach offers several advantages such as less pain, and lower risk of ethanol leakage into surrounding tissues and, owing to collapse of the cyst after drainage of its liquid content, this small amount of ethanol is sufficient to contact with the wall without dilution. On the other hand, we decided not to re-aspirate the ethanol after it was instilled into the empty cyst cavity. Previous studies reported similar results in the non-re-aspiration and re-aspiration groups [22]; however, the non-re-aspiration technique is less time consuming and reduces the number of injections in each patient, and minimizes the possibility of the needle protruding from the cyst cavity due to longer handling during the re-aspiration phase of the procedure. The efficacy of the procedure in reducing of cyst volume and symptoms in the present study supports this approach.

A major concern in all interventional procedures is patient comfort. We specifically tested pain sensation during PEIT using a visual scale, and it is noteworthy that over 80 % of patients reported virtually absent or mild pain and none of the treated group subjects described it as greater than moderate. Previous reports described a transient mild or burning sensation, which was more intense in the largest cysts [7, 24]. Based on these results no local anaesthesia is usually recommended. In our protocol, the injection of a small amount of lidocaine into the cyst cavity before ethanol instillation was included. The high rate of patients reporting virtually no pain (almost 40 %) supports continuing with this procedure.

There is a lack of information in the literature regarding the quality of life of patients with recurrent thyroid cysts treated with ethanol [8]. We addressed this question using a general health questionnaire (SF 36) and a specific questionnaire for symptoms of goitre, we detected normal health quality status as reflected in the SF-36 results and a significant improvement in goitre symptoms using the specific questionnaire. We tested general health only after performing PEIT since, in thyroid nodules with no associated hypo- or hyperthyroidism, the main complaint is local neck symptoms. Post-treatment evaluation confirmed that the procedure did not affect the patient well-being, which therefore had to be similar to that of the general population in terms of SF-36 score. A summary of physical and mental scores showed good status in these patients compared the normal population. Moreover, the specific symptoms of goitre improved dramatically until its disappearance in many patients. To our knowledge, no previous studies had been conducted on QoL in patients with ethanol-treated thyroid cysts and our results add new data in favour of PEIT as an alternative to thyroid surgery. The American and European guidelines [2, 21] recommend PEIT for thyroid cysts, but only those that recur after repeated aspirations. Based on our results and previous reports [6, 7, 16, 19, 22, 23], we believe PEIT should be included as the treatment of choice after the first unsuccessful drainage of cysts. Although the protocol for PEIT is not universally unified regarding the amount of alcohol instilled, re-aspiration of the instilled ethanol and the different use of anaesthesia according to groups, the general results obtained by PEIT are not affected by these protocol differences. Thus, albeit not mandatory, it would be convenient to have a homogeneous protocol established by scientific societies and ad hoc guidelines based on the reported results and local experience [25, 26].

## Conclusions

PEIT in recurrent thyroid cysts appears to be a highly efficacious and safe technique. The improvement in clinical conditions obtained with very low pain sensation, absence of complications and good QoL perceived after the procedure render PEIT an optimum and more recommendable alternative to thyroid surgery. In our opinion, future guidelines should include PEIT prior to surgical treatment and establish a uniform consensus protocol.
